# Host-directed kinase inhibitors act as novel therapies against intracellular *Staphylococcus aureus*

**DOI:** 10.1038/s41598-019-41260-8

**Published:** 2019-03-19

**Authors:** Natalia Bravo-Santano, Helen Stölting, Frederic Cooper, Narina Bileckaja, Andrea Majstorovic, Nadine Ihle, Luis M. Mateos, Yolanda Calle, Volker Behrends, Michal Letek

**Affiliations:** 1 Health Sciences Research Centre, University of Roehampton, London, UK; 2 Department of Molecular Biology, Area of Microbiology, University of León, León, Spain

## Abstract

Host-directed therapeutics are a promising anti-infective strategy against intracellular bacterial pathogens. Repurposing host-targeted drugs approved by the FDA in the US, the MHRA in the UK and/or regulatory equivalents in other countries, is particularly interesting because these drugs are commercially available, safe doses are documented and they have been already approved for other clinical purposes. In this study, we aimed to identify novel therapies against intracellular *Staphylococcus aureus*, an opportunistic pathogen that is able to exploit host molecular and metabolic pathways to support its own intracellular survival. We screened 133 host-targeting drugs and found three host-directed tyrosine kinase inhibitors (Ibrutinib, Dasatinib and Crizotinib) that substantially impaired intracellular bacterial survival. We found that Ibrutinib significantly increased host cell viability after *S. aureus* infection via inhibition of cell invasion and intracellular bacterial proliferation. Using phosphoproteomics data, we propose a putative mechanism of action of Ibrutinib involving several host factors, including EPHA2, C-JUN and NWASP. We confirmed the importance of EPHA2 for staphylococcal infection in an *EPHA2*-knock-out cell line. Our study serves as an important example of feasibility for identifying host-directed therapeutics as candidates for repurposing.

## Introduction

*Staphylococcus aureus* is a facultative intracellular pathogen carried by about 40% of the population in their nasal passages^1^. During nasal colonization, *S. aureus* is capable of internalizing into human nasal epithelial cells^2^, and the colonization of the anterior nares increases the risk of developing bacteremia in persistent carriers^3^. In addition, intracellular *S. aureus* is associated with recurrent rhinosinusitis^2,4^, tonsillitis^5^ and chronic osteomyelitis^6^. Furthermore, host cell invasion and intracellular survival could be used by *S. aureus* to infect macrophages, spread to secondary points of infection, evade immune recognition, and avoid exposure to last-resort antibiotics such as vancomycin^7^. This is particularly important for hospital-acquired infections with the methicillin-resistant *S. aureus* (MRSA). Therefore, novel anti-infective strategies are urgently needed against this versatile pathogen to complement traditional antibiotherapy.

As part of their host-defense evasion mechanism, intracellular pathogens subvert and exploit a wide range of host factors and pathways to support their intracellular survival^8^, targeting multiple pathways to assure their intracellular proliferation^9^. Hence, the study of host-pathogen interactions may lead to the identification of potentially novel pathogen-specific drug targets and/or host-directed therapeutics.

Host-directed approaches could: (i) interfere with the host-pathways exploited by intracellular pathogens to survive within the host cell, (ii) enhance the immune response by stimulating host-defense responses against intracellular infection, (iii) target those pathways that cause hyper-inflammation and (iv) adjust host factors that lead to unstable responses at the site of infection^10^. Specifically, host-directed strategies comprise a range of different therapeutic agents such as monoclonal antibodies, vitamins, cytokines, cellular therapy, recombinant proteins and repurposed drugs^11^.

Repurposing commercially available drugs that may target host-pathways hijacked by intracellular pathogens is a particularly important strategy. The main advantage of using repurposed drugs is that they show minimal toxicity to the host-cell and they have already been approved for other clinical purposes, which would significantly reduce the necessary time to have these drugs in the market^12,13^.

There are currently several repurposed drugs that are in preclinical phase trials to treat bacterial and viral infections. For instance, Dasatinib – a tyrosine kinase inhibitor – inhibits the replication of Dengue virus via blockade of host proto-oncogene kinase FYN^14^. Imatinib – an inhibitor of BCR-ABL tyrosine kinase – reduces bacterial load and pathology in mouse lungs infected with *Mycobacterium tuberculosis*^15^. In addition, cholesterol biosynthesis inhibition has shown potential against *S. aureus* since it is needed for the internalization of the bacteria and the activation of virulence factors^12,16^.

We recently discovered and characterized how host-autophagy is induced by MRSA through activation of the AMPK pathway. In our study, we showed a significant reduction of intracellular bacterial load in both primary and established cell lines due to host-directed AMPK inhibition of dorsomorphin^17^. Accordingly, mice treated with an autophagy inhibitor are protected from MRSA pneumonia^18^. Based on these observations, here we screened for host-directed drugs that have already been approved for other clinical purposes, seeking to identify novel host-targeted compounds to control the cell infection caused by *S. aureus*.

## Results

### Developing a high-throughput screening for host-targeted therapeutics against intracellular *S. aureus*

**Figure 1:**
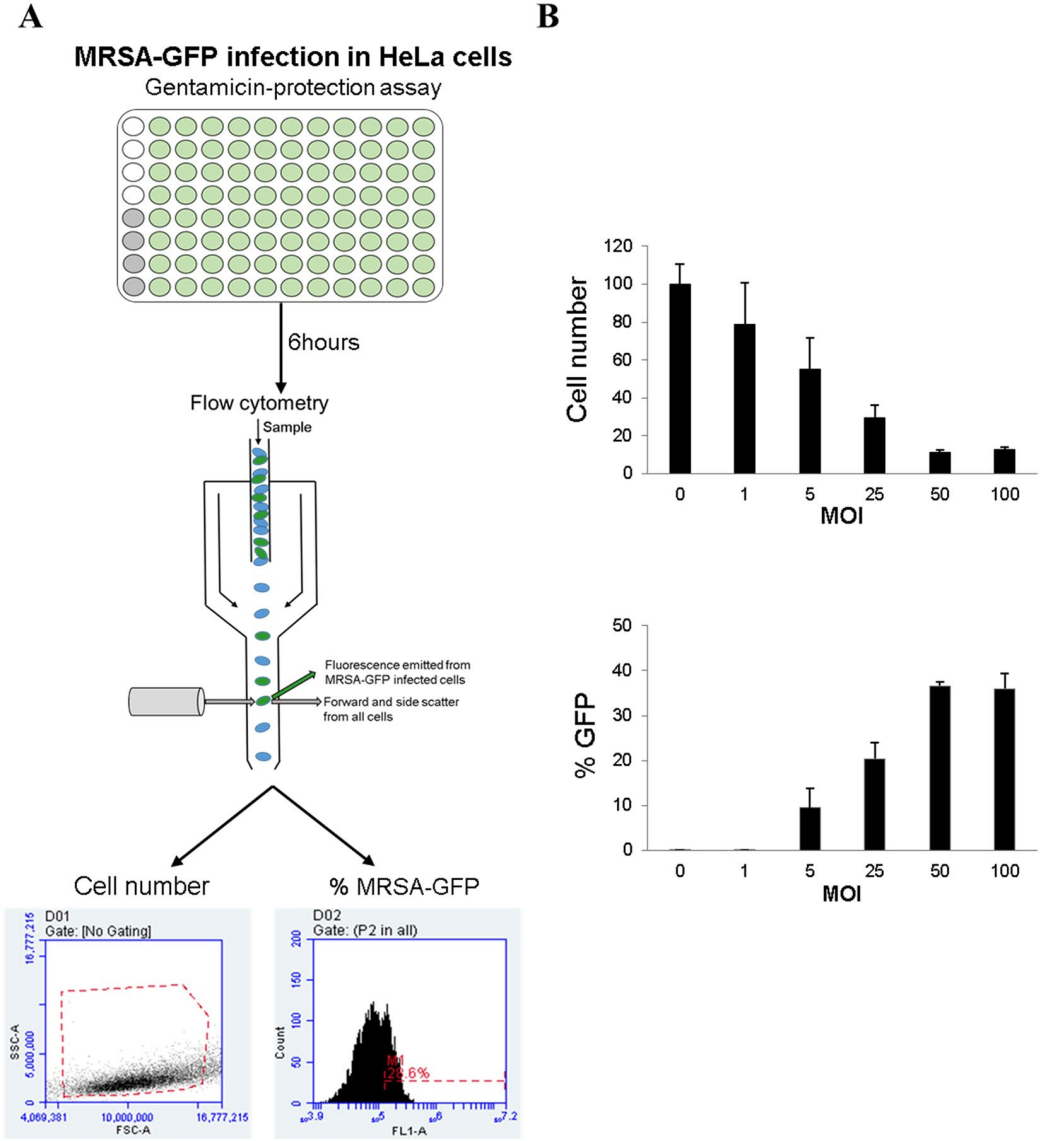
Host-directed drug-screening approach and standardization. (**A**) Experimental design of the drug screening approach. Untreated samples were always included as internal controls in each plate: four uninfected (white circles) and four MRSA-infected (grey circles). Each drug was added in a different well (green circles). After 6 hours of infection, samples were analyzed in the flow cytometer where cell number and percentage of cells containing *S. aureus*-GFP were quantified for every sample. (**B**) This approach was standardized using different MOIs. Data are expressed as mean ± standard errors (SE) of three independent experiments performed in duplicates.

**Figure 2:**
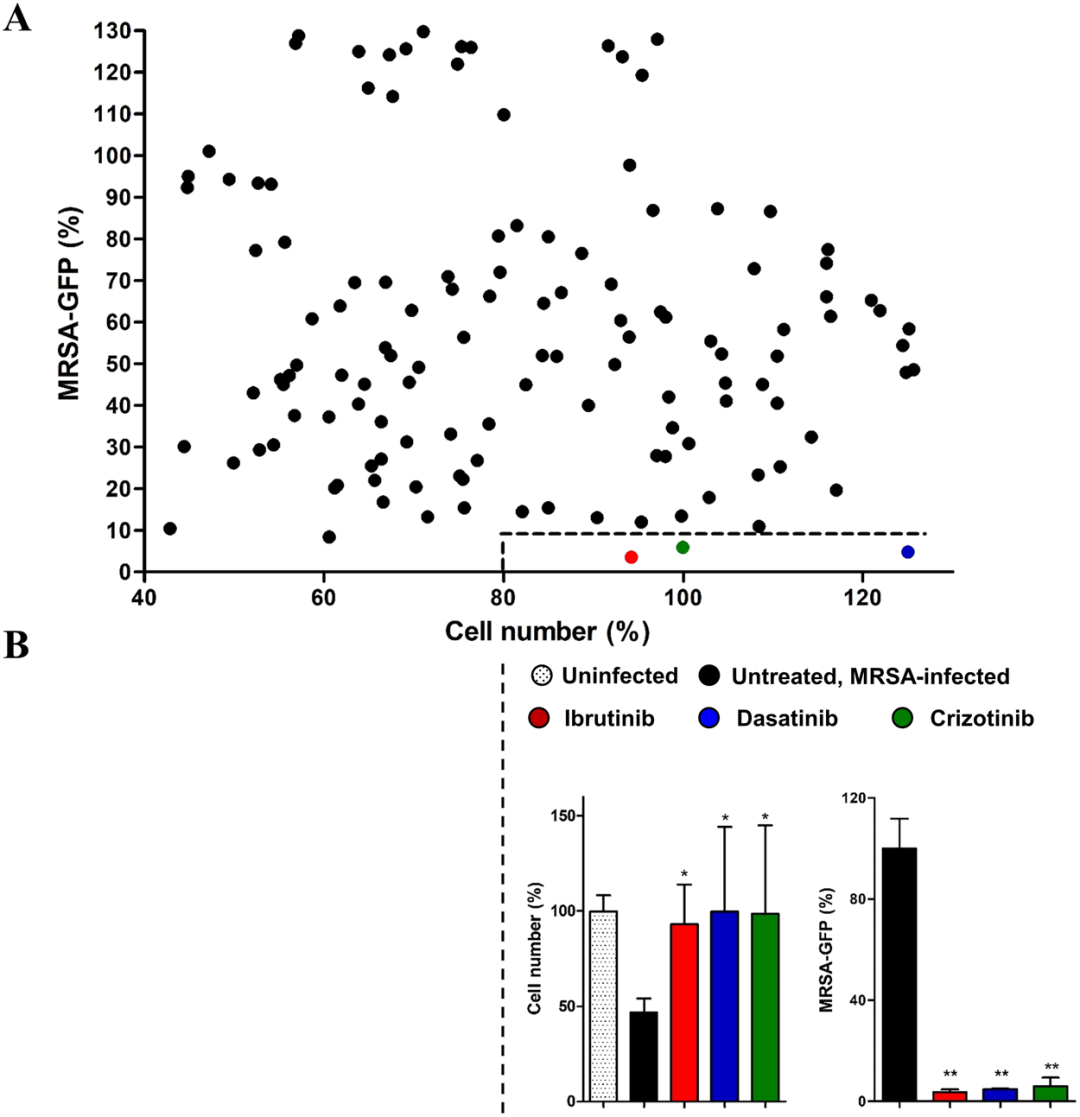
Host-directed drugs screening in HeLa cells after *S. aureus* infection. HeLa cells were infected with *S. aureus* USA300-GFP (MOI 100; 6 hours) in the presence of different drugs (10 µM). (**A**) Host cell viability and percentage of USA300-GFP was measured by flow cytometry and normalized to uninfected cells and untreated *S. aureus*-infected cells, respectively. Each dot represents a different treatment. The dashed line in the right bottom corner depicts the established cut-off. (**B**) Percentage of cell viability and *S. aureus*-GFP for the three drugs within the cut-off values. Means ± standard errors (SE) of three independent experiments are shown. One-way ANOVA and multiple comparison Tukey’s tests were performed to validate the statistical significance across conditions. p-value ≤ 0.05*; ≤0.01**.

To systematically screen a large number of drugs for their activity against staphylococcal invasion, we developed a fluorescence-based high-throughput assay (Fig. 1). The assay uses flow cytometry to quantify viable host cells expressing the red fluorescent protein mCherry while also counting infected cells with bacteria constitutively expressing the green fluorescent protein (GFP). After establishing the scalability of the approach (Fig. 1B), we used a length of infection of 6 hours with a multiplicity of infection (MOI) of 100 to screen the effect of 133 host-targeting FDA-approved drugs (Approved Oncology Drugs Set VIII – National Cancer Institute, US) on host and bacterial cell viability (Fig. 2).

Using a two-fold cut-off of a 90% reduction of intracellular MRSA compared to the untreated host cells and a host cell viability of at least 80% compared to uninfected host cells, we identified three drugs meeting these criteria: Ibrutinib, Dasatinib and Crizotinib (Fig. 2B; for results of all drugs, see Table S1). Of these three, Ibrutinib induced the clearest inhibitory effect on MRSA survival and the lowest variability across experimental replicates, therefore we selected this drug for further validation and downstream experiments.

### Ibrutinib halts host cell invasion and impairs intracellular survival of *S. aureus*

To test whether the observed effect of Ibrutinib on host cell infection by *S. aureus* was due to host-pathway inhibition or a direct effect on bacterial growth, we measured bacterial growth curves in the presence and absence of Ibrutinib. We did not find any significant differences in *S. aureus* growth in the presence of Ibrutinib compared to growth in DMEM only, indicating that the previous observations resulted from a host-directed effect (Fig. S1).

**Figure 3:**
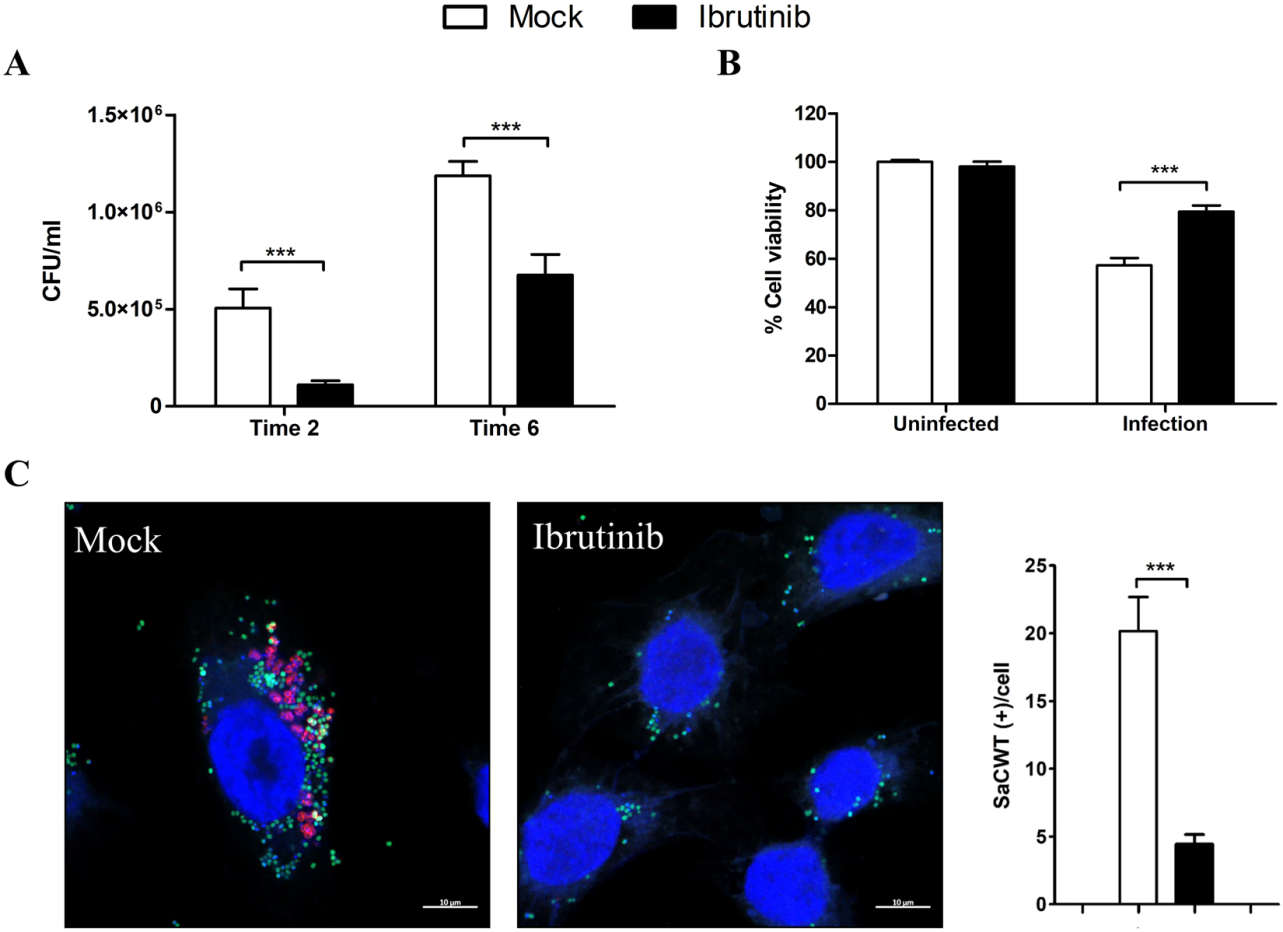
Ibrutinib treatment during *S. aureus* infection increases host cell viability whereas intracellular survival and phagosomal escape of *S. aureus* to host cell cytosol is hampered. HeLa cells were infected with *S. aureus* USA300 (MOI 100) in the presence of DMSO (Mock) or 10 µM of Ibrutinib. (**A**) Intracellular MRSA survival was quantified by colony forming unit (CFU) counting after 2 and 6 hours of infection. (**B**) Host cell viability was quantified by flow cytometry, using a double annexin V-FITC and PI staining after 6 hours of infection. Cell viability was normalized by the percentage of uninfected and untreated cells. (**C**) HeLa cells expressing the phagosomal escape reporter mCherry-CWT were infected with *S. aureus* USA300-GFP (MOI 100) for 6 hours. DAPI was employed for nucleus staining and images were taken using confocal microscopy. Bar indicates 10 µm. Quantification refers to the number of bacteria positively stained with mCherry-CWT per cell. Data are expressed as means ± standard errors (SE) of three independent experiments performed in duplicates. Two-way ANOVA following by Bonferroni *post hoc* tests were performed to test for statistical significance across conditions. p-value ≤ 0.001***.

To validate cell viability readings based on mCherry expression, we measured markers that are activated upon cell death (annexin-FITC and propidium iodide) and found again a significant increase in cell viability of *S. aureus*-infected cells that were treated with 10 µM of Ibrutinib in comparison to untreated cells (Fig. 3B). We also found that recovery of intracellular *S. aureus* after both 2 and 6 hours post-infection was significantly lower in the presence of Ibrutinib (Fig. 3A). Specifically, the reduction of intracellular MRSA was more pronounced at early times of infection (2 hours), suggesting that the drug’s effect is particularly important for cell internalization.

Cytoplasmic replication of *S. aureus* upon phagosomal escape is considered one of the last steps of successful cell invasion^19^. Therefore, we quantified the number of cytosolic bacteria per cell using a phagosomal escape reporter (C-terminal cell wall-targeting domain (CWT) of lysostaphin^17^) and found a fourfold reduction in bacterial numbers in the cytosol in the presence of 10 µM of Ibrutinib (Fig. 3C).

### Ibrutinib’s effect on *S. aureus* intracellular infection is likely to be BTK-independent

Ibrutinib is a potent inhibitor of the Bruton’s tyrosine kinase (BTK) pathway in B cells^20^. However, BTK is mainly expressed in cells of the immune system^21^, and a number of alternative protein kinases that are targeted by Ibrutinib have been recently identified, e.g. EGFR, ITK or TEC^22^. To test the putative role of BTK in our model, we evaluated the effect on intracellular *S. aureus* survival of another two BTK inhibitors, Acalabrutinib and ONO-4059^23^. We did not detect significant differences in host cell viability or bacterial intracellular survival for these compounds (Fig. S2), suggesting a BTK-independent mechanism of action due to an off-target effect of Ibrutinib in our model of infection.

### Phosphoproteomics of *S. aureus*-infected cells in response to Ibrutinib identifies candidate pathways

**Figure 4:**
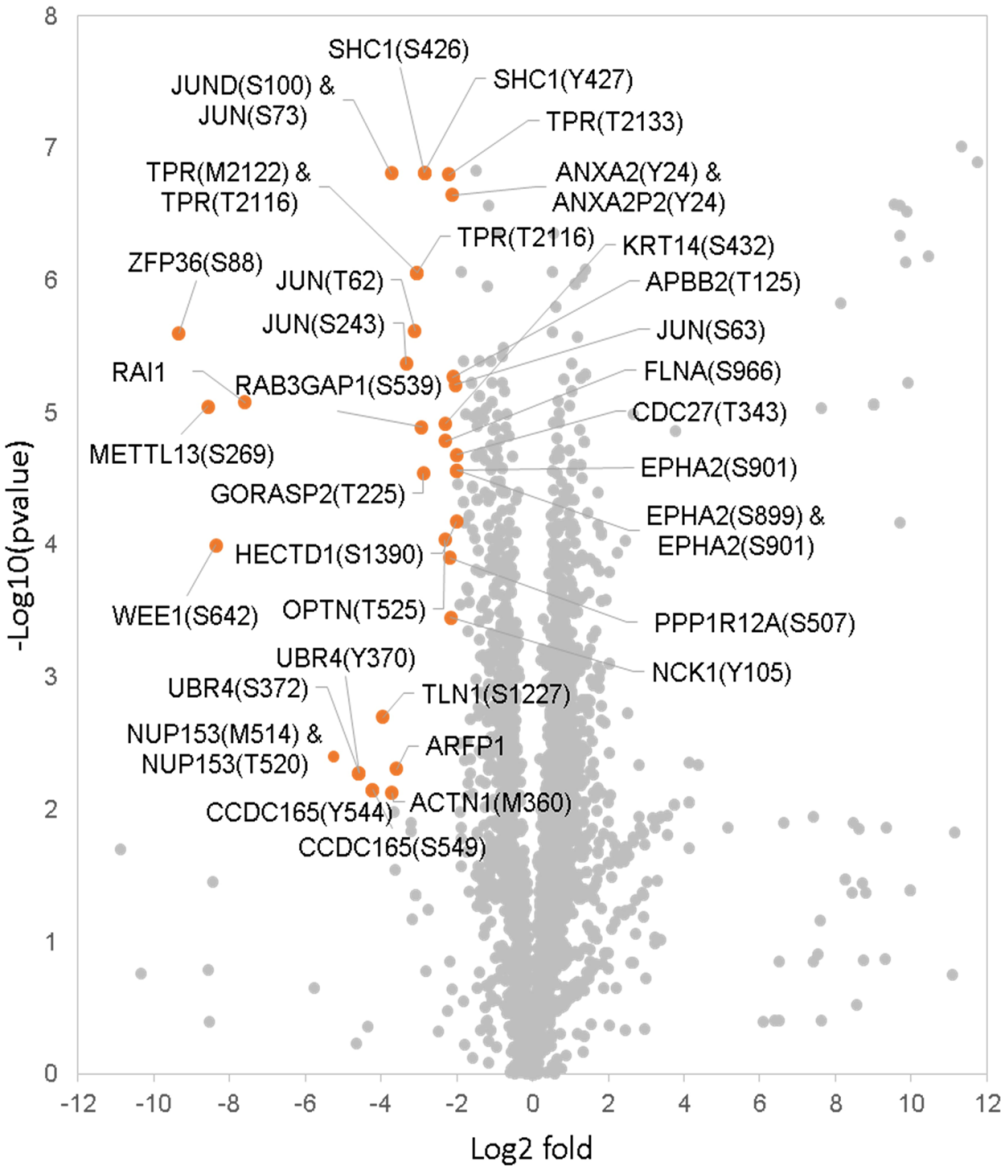
Phosphoproteome of *S. aureus*-infected HeLa cells under Ibrutinib treatment. Log2 Fold change is plotted against the −Log10 p-value of each phosphopeptide under Ibrutinib treatment in relation to the untreated control. Orange dots comprise phosphopeptides whose Log2 fold was lower than −2 and −Log10 p-value was higher than 2.

To elucidate the putative mechanism of action of Ibrutinib in our model, we performed phosphoproteomics analyses in *S. aureus*-infected cells in the presence and absence of the drug. We found that the phosphoproteome of MRSA-infected cells was clearly affected by the treatment with Ibrutinib (Figs 4, S3 and Table S2). Moreover, although some variability was observed across samples, biological and technical replicates were highly reproducible (Fig. S3). Ibrutinib is an inhibitor of tyrosine (Y) kinases; however, as an off-target effect of this drug may play a role during *S. aureus* infection, we also included peptides with phosphoserine/threonine (S/T) sites in our analyses. We focused our analyses on the most significant hits, and 34 proteins were identified when applying a strict cut-off (Log2 fold change ≤−2 and Log10 p-value ≥ 2; Fig. 4). Of these, several proteins could control host pathways that could be crucial during cell infection. Our hits included several phosphopeptides of the JUN and JUND transcriptional factors of the AP-1 complex, which are at the crossroads of different signalling pathways required for proliferation, survival or apoptosis^24^. In addition, we identified ANXA2, a multifaceted protein that is involved in intracellular membrane repair, among many other processes^25^; RAB3GAP1 modulates autophagosomal biogenesis^26^; FLNA has different roles on cytoskeleton remodelling^27^. Finally, EPHA2 stimulates the MAP/ERK kinase signalling cascade through SHC1^28^, which controls the expression of several genes involved in cell cycle.

**Figure 5:**
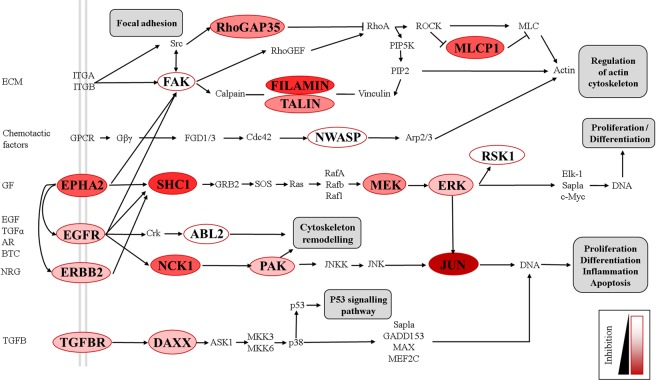
Effects of Ibrutinib on the phosphoproteome of *S. aureus*-infected HeLa cells. The genes for which we detected a modification in their phosphorylation levels are displayed in capitals and surrounded by a red line. The level of inhibition of each gene under Ibrutinib treatment is represented by the intensity of the red-colour within the circle as shown by the legend. Different pathways that are downstream affected by the inhibition of these genes are illustrated in grey-squares.

To further explore the phosphoproteome of *S. aureus*-infected cells under Ibrutinib treatment, we performed a functional pathway analysis by using DAVID Bioinformatics Resources 6.8^29^. For this, we selected phosphopeptides with p-values < 0.05 and Log2 fold change ≤−1.1. The resulting 158 phosphopeptides were analysed to investigate the host pathways affected by the treatment, which revealed that Ibrutinib mainly influenced five cellular pathways in infected host cells: (i) focal adhesion, (ii) ERBB and (iii) MAPK signalling pathways, (iv) adherens junctions and (v) the regulation of actin cytoskeleton (Table S3). Twenty proteins were commonly implicated in these five cellular pathways (Table S4 and Fig. 5), including EPHA2, EGFR and ERBB2.

Finally, we performed a kinase substrate enrichment analysis (KSEA^30^). KSEA is a powerful tool to estimate kinase activity based on the collective phosphorylation status of its substrates. Using this analysis, we identified several kinases that belong to MEK/ERK pathway (MEK2, ERK1/2/7, RSK2 and p90RSK) and three c-Jun N-Terminal kinases (JNK1/2/3; Fig. S4), which reflects the importance of MEK/ERK/c-JUN signalling for intracellular *S. aureus* survival and proliferation. Conversely, none of our analyses flagged up BTK as a target in our model of infection, further pointing towards a BTK-independent mechanism of action for Ibrutinib in this context.

### Downstream analysis reveals the importance of EPHA2 for staphylococcal invasion

To validate our results and to further characterize the host cell response to *S. aureus*, we investigated expression and phosphorylation levels of three selected proteins that may be involved in the mechanism of action of Ibrutinib. We initially studied the phosphorylation patterns of two proteins that were identified before as important host factors for intracellular *S. aureus*: N-WASP and C-JUN^31,32^. We have then focused our attention on EPHA2, a host receptor that has not been studied before in this context.

Interestingly, we identified N-WASP as a potential target of the Ibrutinib effect on MRSA-infected cells. *S. aureus* triggers actin comet-tails through N-WASP phosphorylation^31^. In addition, the intracellular pathogen *Shigella flexneri* promotes BTK expression and N-WASP phosphorylation for its intracellular dissemination^33^. Accordingly, the total protein levels of N-WASP were markedly increased after *S. aureus* infection, and these levels were lowered in infected cells treated with Ibrutinib (Fig. S5). However, the N-WASP/phospho-N-WASP ratio was not significantly affected by Ibrutinib in MRSA-infected cells (Fig. S5), suggesting an indirect effect of Ibrutinib on N-WASP phosphorylation levels, and ruling out again an implication of BTK.

**Figure 6:**
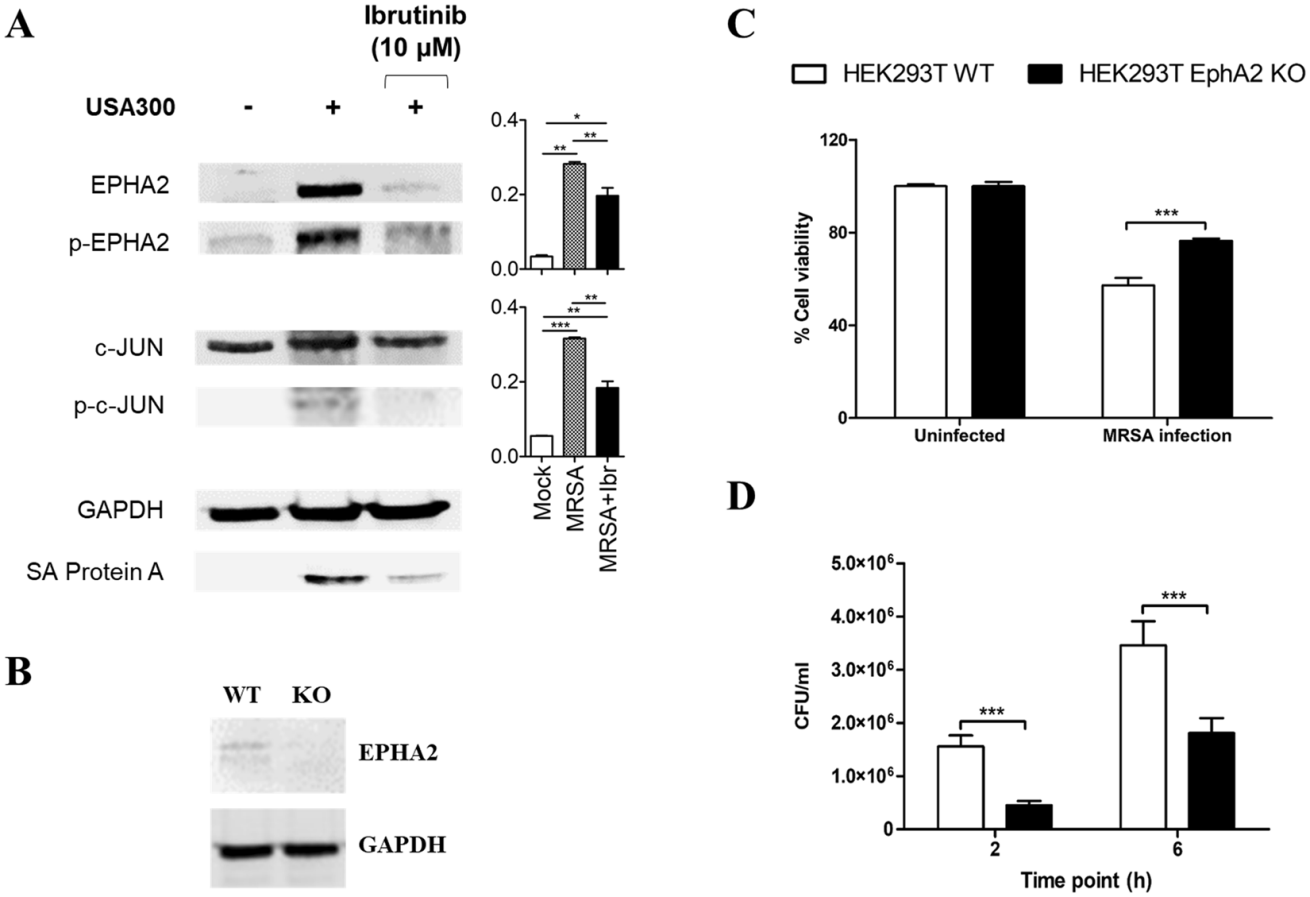
Phosphoproteomics validation reveals the importance of host-membrane receptor EPHA2 during intracellular *S. aureus* infection. (**A**) HeLa cells were infected with *S. aureus* USA300 (MOI 100) for 6 hours in the presence or absence of Ibrutinib. Protein lysates were analysed by Western-blot against EPHA2, p-EPHA2, c-JUN and p-c-JUN. GAPDH antibody was employed as loading control. Protein A from *S. aureus* was also detected and included as an internal control of the *S. aureus* infection. The cropped blots are used in the figure, and full-length blots are presented in Fig. S8. Basal and phosphorylated levels were quantified for EPHA2 and c-JUN proteins and the ratio of phosphorylated/basal levels was calculated for both proteins. Data are expressed as means ± standard errors of two independent experiments. (**B**) Protein lysates of HEK293T wild type and *EPHA2* knockout cells were analysed by Western-blot against EPHA2, using GAPDH antibody as loading control. The cropped blots are used in the figure, and full-length blots are presented in Fig. S9. (**C**,**D**) HEK293T wild type and EPHA2 knockout cells were infected with *S. aureus* USA300 (MOI 100) for 6 hours. (**C**) Host cell viability was quantified by flow cytometry, using a double annexin V-FITC and PI staining. Cell viability was normalized by the percentage of uninfected cells. (**D**) Intracellular MRSA survival was quantified by colony forming units (CFU) counting after 2 and 6 hours of infection. Data are expressed as means ± standard errors of three independent experiments performed in duplicates. One-way ANOVA following by Tukey´s multiple comparison test (6A) and Two-way ANOVA following by Bonferroni *post hoc* test (6C&D) were performed to validate statistical significance across conditions. p-value ≤ 0.05*; ≤ 0.001***.

In concordance with our phosphoproteomics data, we observed that phosphorylation levels of c-JUN were downregulated in infected cells treated with Ibrutinib (Fig. 6A). Furthermore, basal levels of c-JUN remained stable among the three conditions, indicating that Ibrutinib directly controls the phosphorylation of this protein (Fig. 6A).

In addition, the basal levels of EPHA2 were markedly increased in *S. aureus*-infected cells compared to the controls and were downregulated by Ibrutinib treatment (Fig. 6A). Interestingly, EPHA2 has been shown to be important in other models of intracellular infections^34^. As inhibitors of Ephrin receptors such as ALW-II-41–27 exhibit cross-reactivity with a number of EPHA and EPHB receptors (e.g. EPHA2/5/8 and EPHB1/2/3^35^), we tested the role of EPHA2 during *S. aureus* cell infection in an *EPHA2* knockout cell line derived from HEK293T^36^. We quantified host cell viability and intracellular bacterial survival and – in agreement with our previous results – found that host cell viability was restored to 80% in the *EPHA2* knockout cell line after 6 hours of *S. aureus* infection when compared to the wild type (Fig. 6B). At the same time, intracellular bacterial load was reduced twofold at 2 and 6 hours post-infection in the *EPHA2* knockout cell line (Fig. 6C), suggesting that the EPHA2 receptor is important for the successful internalization of *S. aureus* inside host cells. Moreover, we also tested the effect of Ibrutinib on HEK293T wt and *EPHA2* knockout cell lines (Fig. S6). As previously observed in HeLa cells, Ibrutinib treatment on HEK293T wild type cells showed a similar pattern: an increased host cell viability after 6 hours of infection and a significant reduction of intracellular *S. aureus* load at both 2 and 6 hours post infection. Regarding *EPHA2* knockout cells, the effect of Ibrutinib on both host cell viability and intracellular bacterial survival was not statistically significant compared to untreated *EPHA2* knockout cells and these findings further support the hypothesis that EPHA2 may be the target of Ibrutinib in our infection model.

## Discussion

In this study, we identified three potential candidates that may contribute to the clearance of intracellular MRSA infection (Ibrutinib, Dasatinib and Crizotinib) from a pool of 133 host-directed drugs. For in-depth characterisation, we focused on Ibrutinib, which caused a significant reduction of intracellular bacterial load at early time points of infection. This suggested that the host-pathway inhibited by Ibrutinib could play an important role at early steps of infection, such as bacterial cell invasion or internalization.

Among all the different host-directed strategies against bacterial and viral pathogens, tyrosine kinase inhibitors have shown potential for clearing intracellular infections and two drugs are already under preclinical trials: Imatinib and Dasatinib^11^. Imatinib – an inhibitor of BCR-ABL tyrosine kinase – restricts *M. tuberculosis* replication within macrophages^37^. Moreover, a recent study showed that low doses of Imatinib reduce bacterial load of *Francisella* spp. by inducing myelopoiesis and thus, the stimulation of host-immune responses by this compound may promote clearance of several microbial pathogens^15^. Imatinib also exhibited promising results in our study but did not meet our stringent cut-off criteria (Table S1).

Dasatinib was one of the most promising candidates from our drug screening. After *S. aureus* infection, host cell viability was highly increased whereas intracellular bacterial load was drastically reduced in the presence of this drug. Dasatinib is a tyrosine kinase inhibitor that targets a wide variety of kinases and receptors including ABL, the SRC family kinases, the receptor tyrosine kinases c-KIT, platelet-derived growth factor receptor (PDGFR), discoidin domain receptor 1 (DDR1), c-FMS, and Ephrin receptors^38^. One of these pathways may be hijacked by MRSA during intracellular infection, providing a potential mechanism for the significant reduction of intracellular bacterial load during Dasatinib treatment.

It has previously been shown that some intracellular pathogens are able to manipulate host actin to drive movement through the cytosol and to promote cell-to-cell spread^39^. Actin maintains tissue integrity by providing structural stability at cell-to-cell junctions and promotes the formation of membrane extensions during cell migration as well as intracellular trafficking^39^. Pathogens can trigger host actin-cytoskeleton modifications in different ways: (i) using secreted toxins that directly target actin or its regulators^40^, (ii) exploiting actin assembly to promote their invasion^41^ or (iii) hijacking the host-actin polymerization machinery to induce intracellular or surface-associated motility.

For the pathogen *Shigella flexneri* it has been suggested that it requires Bruton’s Tyrosine Kinase (BTK)-mediated phosphorylation of N-WASP to facilitate actin-based motility and dissemination^42^. Based on these observations and since Ibrutinib is used as a BTK inhibitor, we initially speculated that this pathway might also be important during *S. aureus* infection. However, the levels of both N-WASP and phospho-N-WASP were decreased in MRSA-infected cells after Ibrutinib treatment, with the N-WASP/phospho-N-WASP ratio not significantly altered across the different conditions. The reduction in the phosphorylation of N-WASP detected by LC-MS/MS is due to a reduction of basal levels of the protein, probably caused by a reduction of intracellular bacterial load.

As mRNA expression of BTK is limited to hematopoietic cells^21^ and some off-target effects of Ibrutinib have already been reported^22^, we hypothesised that the effect of Ibrutinib is independent of its action on BTK. To confirm, we tested two other BTK’s inhibitors – Acalabrutinib and ONO-4059 – and found no significant effects on either host cell viability or *S. aureus* intracellular survival.

Interestingly, Berglöf *et al*. (2015) reported novel targets of Ibrutinib that are well expressed in epithelial cells, such as the Epithelial Growth Factor Receptor family members EGFR, ERBB2 and ERBB4^22^. Hence, these receptors might represent the potential targets of Ibrutinib during MRSA cell infection.

Our analysis revealed that the phosphoproteome of *S. aureus*-infected cells was clearly affected by Ibrutinib treatment. Although we found numerous phosphopeptide changes between treatment and control, functional analyses allowed us to focus on several affected pathways. Many of the genes that were inhibited by Ibrutinib are related to cytoskeleton rearrangement. For example, filamins (FLNA and FLMNB) are actin-binding proteins that stabilize actin filament networks and are important for cell locomotion^43^. In addition, ABL-family kinases are essential regulators of host cytoskeleton. They translate extracellular signals into cytoskeleton rearrangements affecting primarily host cell motility. They are also able to control other kinases, such as Rho and Rac GTPases, to stimulate assembly of protein complexes that activate the formation of actin filaments by the ARP2/3 complex^44^. All of these proteins were inhibited under Ibrutinib treatment after *S. aureus* cell infection (Fig. 6). Similarly, a treatment with the inhibitor of actin polymerization cytochalasin D blocks internalization of *S. aureus* in mammalian cells^45^.

In addition, it has previously been shown that many of the proteins that were inhibited by Ibrutinib in our study also play a role in other viral or bacterial infections. Rho-GTPases are involved in actin cytoskeleton remodelling and are manipulated by the HIV-1 envelope protein to facilitate viral entry into the host cell^46^. The MAPK signalling pathway is one of the most prominent host-pathways hijacked by intracellular pathogens^47^. Specifically, *M. tuberculosis* activates host p38-MAPK pathway in order to inhibit CD1 expression and thus, subverts part of the host-immune response^48^. Additionally, internalization of *S. aureus* has been associated with the activation of focal adhesion kinase (FAK) and SRC-mediated cortactin phosphorylation^49^ and this pathway was also inhibited in the presence of Ibrutinib during MRSA infection. Moreover, kinase substrate enrichment analyses (KSEA; Fig. S4) identified several ERK and c-JUN protein kinases (JNK) as part of the pathways inhibited by Ibrutinib during MRSA infection. Accordingly, internalization of *S. aureus* is impaired when using ERK or JNK inhibitors in fibroblasts and macrophages, respectively^32,50^.

Finally, we have identified several host membrane receptors that could play important roles during *S. aureus* infection, e.g. Ephrin type-A receptor 2 (EPHA2), Epidermal growth factor receptors (EGFR or ERBB1 and ERBB2) and transforming growth factor beta receptor (TGFBR). Moreover, as mentioned above, EGFR and ERBB2 have been previously described as off-targets suspects of the drug Ibrutinib^22^. Links between EGFR and the ephrin-receptor EPHA2 have already been reported^51^. In addition, ADAM10 controls EGFR signalling, which in turn regulates EPHA1/2 signalling complexes^52^. Coincidentally, ADAM10 protein expression is required for the activity of *S. aureus* alpha-hemolysin (Hla) on host cells^53^. Thus, the EPHA2 basal levels may be indirectly controlled by Hla during *S. aureus* cell infection.

Internalization into the host cell is a crucial step to successfully establish infection for intracellular pathogens. Recently, the Ephrin type-A receptor 2 (EPHA2) has been highlighted as a potential common host receptor for the intracellular internalization of many different pathogens^34^. The implication of this tyrosine kinase receptor in infection has already been demonstrated for intracellular bacteria such as *M. tuberculosis*^54^, *Chlamydia trachomatis*^55^ and enteropathogenic *Escherichia coli*^56^. Therefore, we speculated that the facultative intracellular pathogen *S. aureus* might similarly exploit this host membrane receptor to facilitate its internalization into host cells.

In accordance with our hypothesis, exposure of EPHA2 knockout cell lines to *S. aureus* resulted in reduced intracellular bacterial load even at early time points of infection. These results suggest that EPHA2 is important for the successful internalization of *S. aureus*. Additionally, the EPHA2 receptor is linked to many other downstream proteins that may also be involved in the intracellular survival or proliferation of *S. aureus*. For instance, EPHA2 receptor activates focal adhesion kinases (FAK)^57^, which has been previously related to the internalization of *S. aureus*^49^. Furthermore, EPHA2 is associated with SHC, ERK and JUN proteins, which phosphorylation levels were altered under Ibrutinib treatment and play different roles in intracellular infections. Therefore, the host-membrane receptor EPHA2 represents a very promising drug-target for intracellular MRSA anti-infectives.

## Future implications

Infectious diseases are still one of the leading causes of mortality worldwide and the emergence of multidrug resistant strains is considered a major threat to modern healthcare. However, a novel drug discovery approach to combat intracellular bacterial infections is emerging and this is focused on identifying and targeting the host molecular and/or metabolic factors hijacked by intracellular pathogens^11,17^. Although research on host-directed approaches is still in early stages, these strategies have a great potential to fight intracellular infections^11^. Future anti-microbial treatment may combine conventional antibiotics with drugs that target bacterial virulence factors and/or host-directed anti-infectives^12^. As a case in point, our results suggest that host proteins such as EPHA2 could be targeted to halt intracellular *S. aureus*. Nevertheless, further research is required to translate these observations into an effective treatment against infections caused by this pathogen.

## Materials and Methods

### Bacterial strains and culture conditions

*S. aureus* USA300 LAC^58^ and *S. aureus* USA300-GFP^59^ strains were cultured in Nutrient Broth (NB) medium (Sigma-Aldrich). For growth in liquid culture, bacterial strains were incubated at 37 °C, with vigorous shaking (300 rpm) in an Incu-shake MIDI 4020 incubator (SciQuip) and for growth on solid medium, Nutrient agar plates (NA; Sigma-Aldrich) were used and incubated in a 37 °C incubator (LMS).

To evaluate bacterial growth curves, *S. aureus* was incubated in 96-well plates (Sarstedt) with DMEM and optical density at 600 nm (OD_600nm_) was measured at different time points using an EL800 Microplate reader (Bio-Tek).

For preinocula preparation, *S. aureus* was grown overnight in 10 ml of NB at 37 °C with shaking (300 rpm) and 0.5 ml of this culture was used to inoculate a flask containing 50 ml of NB (dilution 1:100). Bacterial cultures were grown until OD_600nm_ of 1 was reached, upon which the culture was centrifuged (4,000 rpm, 15 min, 4 °C). Pellets were then washed twice with Dulbecco’s Phosphate-Buffered Saline (PBS; Sigma-Aldrich), resuspended in 1.5 ml of PBS supplemented with 20% glycerol and aliquots of 100 µl were stored at −80 °C until further use. Concentration of each preinocula was calculated by serial dilution plating and colony forming units (CFU) counting.

### Cell lines and culture conditions

HeLa cells (ECACC 93021013) and HEK293T cells (kindly supplied by Prof Richard M Longnecker^36^) were grown in 100 mm cell culture plates (Sarstedt) with Dulbecco´s Modified Eagle’s medium (DMEM, Gibco) containing pyruvate, glucose and glutamine and supplemented with 10% heat-inactivated foetal bovine serum (FBS, Gibco) and 5% of penicillin and streptomycin solution (Gibco), unless otherwise specified. The cell lines were incubated at 37 °C and 5% of CO_2_ in a Heraeus^®^ BB15 incubator (Thermo Scientific).

### Intracellular infection assays

For host-cell viability and immunofluorescence assays, HeLa and HEK293T cells were seeded in 24-well plates (Sarstedt) in DMEM without antibiotics and at cell density of 7.5 × 10^4^ cells per well. For the drug-screening, HeLa cells were seeded in 96-well plates at cell density of 1.8 × 10^4^ cells per well. In all cases, cells were allowed to settle overnight at 37 °C, in 5% CO_2_.

The next day, an aliquot of bacterial preinocula was thawed, diluted in 900 µl of PBS and centrifuged at 4,000 rpm for 5 min. The pellet was washed twice with PBS prior to addition of DMEM without antibiotics at a bacterial density that corresponds to a multiplicity of infection (MOI) of 100. Then, the bacterial suspension was added to each well, plates were centrifuged immediately at 1,500 rpm for 5 min and incubated at 37 °C in 5% CO_2_ for 45 min to allow bacterial internalization. Medium was then replaced by DMEM supplemented with 100 µg/ml gentamycin to kill extracellular bacteria, and the plates were placed back in the incubator until the desired time points were reached.

### Host cell viability assay

Samples for host cell viability quantification were collected after 6 hours of infection. To recover both necrotic and apoptotic cells, supernatants of each well were transferred into clean Eppendorf’s tubes (Fisher Scientific). Cells were trypsinized for 5 min, diluted in DMEM without antibiotics, mixed with the supernatants, and centrifuged at 1,500 rpm for 10 min. Afterwards, cells were double stained with annexin V-FITC and propidium iodide according to manufacturer’s recommendations (Becton Dickinson, BD), diluted in 50 µl of DMEM per sample and incubated for 15 min at room temperature. Stained cells were centrifuged at 1,500 rpm for 10 min, supernatant was aspirated, and cell pellets were fixed using 150 µl of BD Cytofix buffer (Becton Dickinson, BD) for 15 min at 4 °C. Finally, samples were diluted with 350 µl of DMEM and host cell viability was measured by flow cytometry (BD Accuri^TM^ C6 Plus).

For drug-screening, a high-throughput protocol was standardized. HeLa cells expressing mCherry were created with a P12-MMP derivative^60^. To produce this vector, mCherry was amplified from P12-MMP-mCherry-LC3 with primers TAGCTAAAGCTTGCCACCATGGTGAGCAAGGGCGAG and TAGCTAGCGGCCGCTTTACTTGTACAGCTCGTCCAT. The DNA amplicon was verified by sequencing, digested with HindIII/NotI, and cloned into P12-MMP to create P12-MMP-mCherry. Hela-mCherry cells were created by transduction with P12-MMP-mCherry as described previously^60^. Infection assays were performed with HeLa-mCherry cells and *S. aureus* USA300-GFP in 96-well plates until the time point of 6 hours post-infection was reached. The plates were then centrifuged at 1,200 rpm for 10 min and the supernatant was discarded, retaining only live cells that were attached to the surface of the well. Afterwards, cells were fixed using 20 µl of BD Cytofix buffer (Becton Dickinson, BD) for 15 min at 4 °C and samples were diluted with 100 µl of DMEM. Cells expressing mCherry after 6 hours of infection were considered viable and were quantified by flow cytometry (BD Accuri^TM^ C6 Plus).

### Intracellular bacterial estimation

During drug screening, intracellular bacterial survival was calculated by flow cytometry by measuring the percentage of green fluorescent cells infected by USA300-GFP (BD Accuri^TM^ C6 Plus).

For the validation of the screening results, cells were lysed at different time points of the intracellular infection assays using 0.1% Triton X-100 diluted in PBS and serial dilutions were plated on NA plates for CFU counting.

### Phagosomal escape quantification by confocal microscopy

HeLa cells expressing the phagosomal escape reporter mCherry-CWT^17^ were seeded on coverslips in 24-well plates (7 × 10^4^ cells per well) and infected with *S. aureus* USA300-GFP (MOI 100). After 6 hours of infection, medium was aspirated, and cells were fixed with 0.5 ml of fresh 4% of paraformaldehyde (Fisher Scientific) for 15 min. Coverslips were then washed twice with PBS and mounted on microscope slides (Thermo Fisher Scientific) using ProLong Gold Antifade mountant with DAPI (Thermo Fisher Scientific) for nuclear staining. Slides were viewed on a confocal microscope ZEISS LSM 800 with Airyscan and images were acquired and processed using Zen Blue Software (Zeiss).

### SDS-PAGE electrophoresis gel and Western-blot

Cells were harvested by adding ice-cold PBS to each well and detached by using a cell scraper (Fisher Scientific). Cell suspensions were transferred to a 15 ml Falcon tube (Sarstedt) and centrifuged (2,000 rpm, 10 min, 4 °C). Cell pellets were washed with ice-cold PBS and immediately placed on ice for cell lysis. Lysis buffer (50 mM Tris, pH 7.5; 150 mM NaCl; 5 mM EDTA; 1% NP40) was reconstituted with protease inhibitor cocktail (Sigma-Aldrich), phosphatase inhibitor cocktail (Sigma-Aldrich) and phenylmethylsulfonyl fluoride (PMSF; Sigma-Aldrich), following manufacturer’s instructions. Cell pellets were resuspended in 50 µl of lysis buffer per well, transferred to Eppendorf’s tubes (Sarstedt) and incubated on ice for 30 min, applying heavy vortexing every 10 min. Then, samples were centrifuged at maximum speed for 5 min at 4 °C and supernatants were transferred to new Eppendorf’s tubes and immediately stored at −80 °C. Protein concentration was quantified using DC Protein Assay Kit (Biorad) and every cell lysate was adjusted to the same concentration.

For SDS-PAGE electrophoresis gel, cell lysates were mixed with 2x Reducing Sample Buffer (100 mM Tris, pH 6.8; 10% β-mercaptoethanol; 4% SDS; 0.3% bromophenol blue; 20% glycerol) and boiled in a QBD4 heat block (Grant) at 100 °C for 5 min. Samples were loaded on polyacrylamide gels using 1x of Running Buffer (25 mM Tris; 20 mM Glycine; 0.05% SDS) and run on a Mini-PROTEAN Tetra electrophoresis system (Bio-Rad) with a constant voltage of 100 V for 90 min using a PowerPac Basic Power Supply (Bio-Rad).

Proteins were transferred onto nitrocellulose membranes (GE Healthcare Life Sciences) using a Mini Trans-Blot cell (Bio-Rad) with 1x Transfer buffer (25 mM Tris; 192 mM Glycine; 20% methanol) and Whatman filter paper (GE Healthcare Life Sciences), following manufacturer’s guidelines. The western blot was run on the Tetra electrophoresis system (Bio-Rad) at a constant voltage of 90 V for two hours. Nitrocellulose membranes were blocked using 5% skimmed milk diluted in Tris buffer saline (TBS) solution (50 mM Tris, pH 7.6; 150 mM NaCl) for 1 hour at room temperature. Once the blocking solution was removed, membrane was washed twice with TBS containing 0.05% of Tween20 (TBS-T) and subsequently incubated overnight at 4 °C with the primary antibody, diluted in TBS-T containing 2% BSA and 0.1% sodium azide (Fisher Scientific). After three washes of TBS-T, the membrane was incubated with the corresponding secondary antibody diluted in 5% skimmed milk/TBS-T for 1 hour at room temperature. The membrane was washed again three times with TBS-T and was developed employing Odyssey® Fc Imaging System (Li-Cor). Images were taken, processed and quantified by using Image Studio Software (Li-Cor).

Primary antibodies were purchased from (i) Santa Cruz Biotechnologies (anti-GAPDH [Reference No. sc-47724], anti-EPHA2 [Reference No. sc-398832]), (ii) 2BScientific (anti-p-EPHA2 [Reference No. 12886]), (iii) St Johns Laboratory (anti-c-JUN [Reference No. STJ99617], anti-p-c-JUN [Reference No. STJ90175]), and (iv) Novus Biologicals (anti-N-WASP [Reference No. NBP1-82512] and anti-p-N-WASP [Reference No. NB600-1169]). Secondary antibodies were purchased from Li-Cor (IRDye 680LT goat anti-mouse [Reference No. 926-68070] and IRDye 800LT goat anti-rabbit [Reference No. 926-32211]).

### Phosphoproteomics of *S. aureus*-infected cells

After 6 hours of MRSA infection, cell extracts were obtained by lysing the cells with 50 µl of lysis buffer (20 mM HEPES (pH 8.0)) buffer containing 8 M urea, 1 mM Na_3_VO_4_, 1 mM NaF, 1 mM β-glycerol phosphate and 2.5 mM Na_2_H_2_P_2_O_7_; Sigma-Aldrich) per well. After lysis buffer was added to the cells, samples were thoroughly vortexed for 5 min and immediately stored at −80 °C. Following sonication, cell lysates were centrifuged at 20,000 g for 10 min and the protein concentration was determined by Bradford analysis.

Protein digestion was performed after diluting cell extracts to 2 M Urea with 20 mM HEPES pH 8.0 by incubating with immobilized TLCK-trypsin (20 TAME units/mg; Sigma-Aldrich) for 16 h at 37 °C. Digestion was then stopped by adding 1% of trifluoroacetic acid (TFA; Sigma-Aldrich). The resultant peptide solutions were desalted using Sep-Pak C18 columns (Waters UK Ltd) following manufacture’s guidelines^61^.

Phosphopeptide enrichment was performed using an Immobilized Metal Ion Affinity Chromatography (IMAC) enrichment protocol as previously described^62^. Phosphopeptides were detected in a Liquid Chromatography Tandem Mass Spectrometry (LC-MS/MS) system as previously reported^61,63^.

Mascot Daemon (v2.2.2; Matrix Science, London, UK) was employed to analyse the LC-MS/MS data, including carbamidomethyl (C) as fixed modification and pyro-glu (N-terminal), oxidation (M) and phosphor (STY) as variable modifications^64^. PESCAL was used to quantify the intensities of peptides present in the database across all samples. PESCAL uses restrictions on the molecular mass, retention time, charge and isotopes distribution to confidently identify the studied phosphopeptides^65^. The resulting quantitative results were then exported to Excel files for further normalization and statistical analysis.

Phosphoproteomics data are expressed as means ± S.D. and plotted as heat-map as well as Volcano plot. Statistical differences in phosphopeptides quantification were assessed by Student´s t-test and adjusted for multiple testing using the Benjamini Hochberg false discovery rate method. R software was used for principal component analysis (PCA). Regarding functional analysis, DAVID Bioinformatic software v.6.8^29^ was used to identify the most important KEGG pathways, and KSEA analysis^30^ were carried out to find out the kinase signalling pathways that were activated or inhibited under Ibrutinib treatment.

### Statistical analyses

Statistical tests and graphs plotting were conducted using GraphPad Prism software and significant differences across treatments were assessed by running ANOVA and *post hoc* multiple comparison tests when required.

## Supplementary information

Supplementary Figures and Tables

Table S2
