# Supplementary material for: Host-directed kinase inhibitors act as novel therapies against intracellular Staphylococcus aureus
Source: Sci Rep. 2019 Mar 19;9:4876. doi: 10.1038/s41598-019-41260-8 (PMC6425000; doi:10.1038/s41598-019-41260-8)
Supplement: Supplementary file 1 — Supplementary Figures and Tables [file 41598_2019_41260_MOESM1_ESM.pdf]

Host-directed kinase inhibitors act as novel therapies against intracellular *Staphylococcus aureus*

Natalia Bravo-Santano[a], Helen Stölting[a], Frederic Cooper[a], Narina Bileckaja[a], Andrea Majstorovic[a], Nadine Ihle[a], Luis M. Mateos[b], Yolanda Calle[a], Volker Behrends[a#], Michal Letek[a#]

SUPPLEMENTARY FIGURES

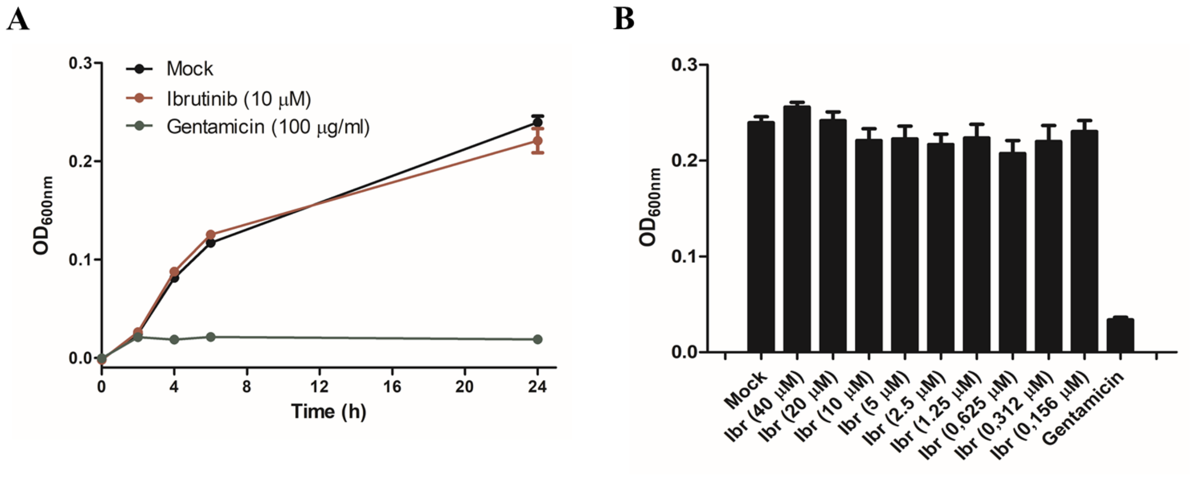

Figure S1. Ibrutinib treatment does not affect *S. aureus in vitro* growth. (A) *S. aureus* USA300 was grown in 96-well plates in DMEM in the presence of DMSO (Mock), Ibrutinib or gentamicin and OD_{600nm} was measured at 2, 4, 6 and 24 hours. (B) Effect of different concentrations of Ibrutinib on USA300 growth after 24 hours in DMEM.

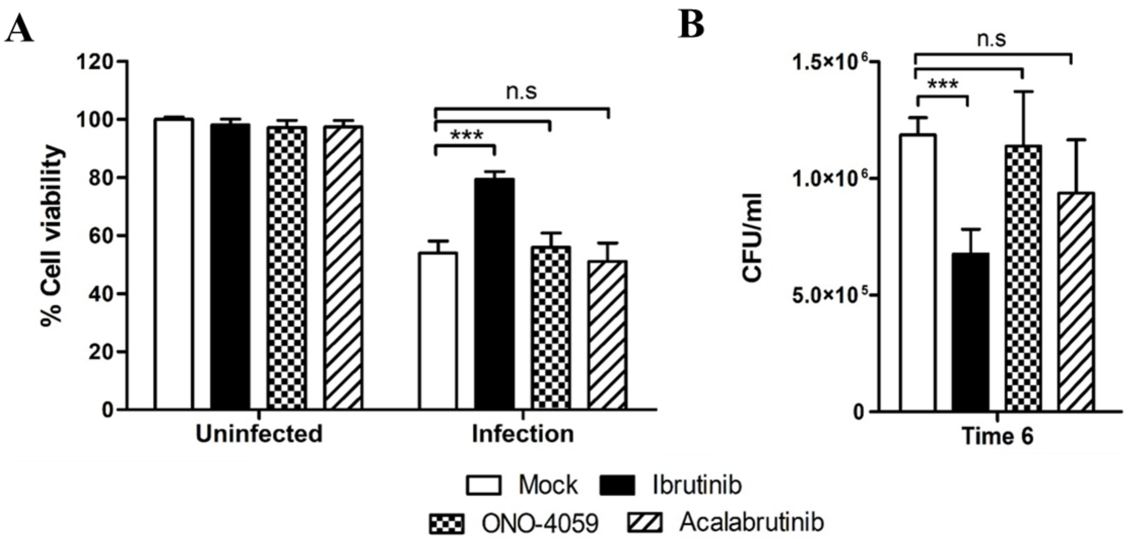

Figure S2. Effect of Btk's inhibitors – Ibrutinib, Acalabrutinib and ONO-4059 – on *S. aureus* infection in HeLa cells. HeLa cells were infected with *S. aureus* USA300 (MOI 100) for 6 hours in the presence of DMSO (Mock), Ibrutinib, Acalabrutinib or ONO-4059. (A) Host cell viability was quantified by flow cytometry, using a double annexin V-FITC and PI staining. Cell viability was normalized by the percentage of uninfected and untreated cells. (B) Intracellular MRSA survival was quantified by colony forming unit (CFU) counting after 6 hours of infection. Data are expressed as means ± standard errors of three independent experiments performed in duplicates. Two-way ANOVA and Bonferroni post hoc tests were performed to validate statistical significance across conditions. p-value ≤0.001 (***), (n.s) no statistical significance.

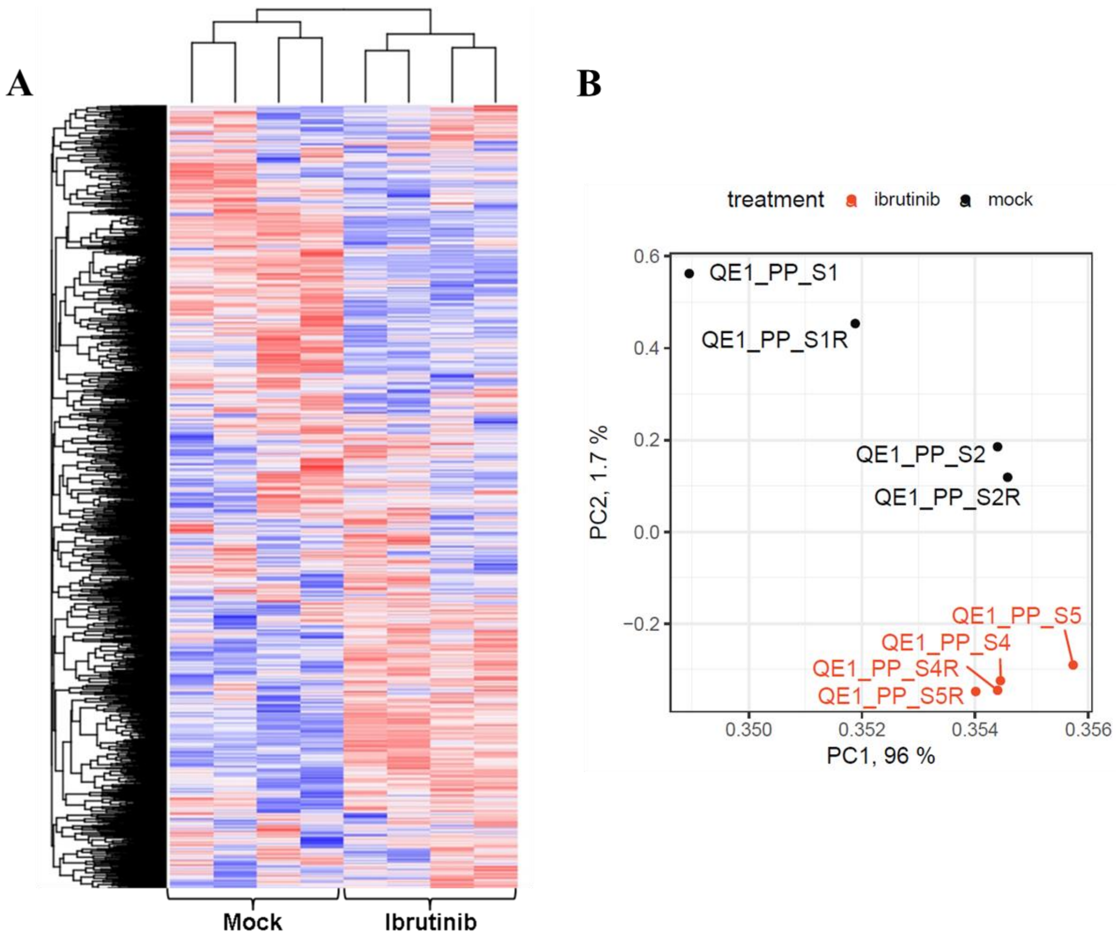

Figure S3. Differences of phosphopeptides levels in *S. aureus*-infected cells under Ibrutinib treatment. (A) Relative values of every phosphopeptide detected through LC-MS was plotted as a Heat-map. Each condition is comprised by four replicates of each treatment: mock (DMSO) and Ibrutinib treatment. (B) Principal Component Analyses (PCA) of the phosphoproteomics data in MRSA-infected cells. Untreated samples are represented in black whereas Ibrutinib-treated samples are displayed in red.

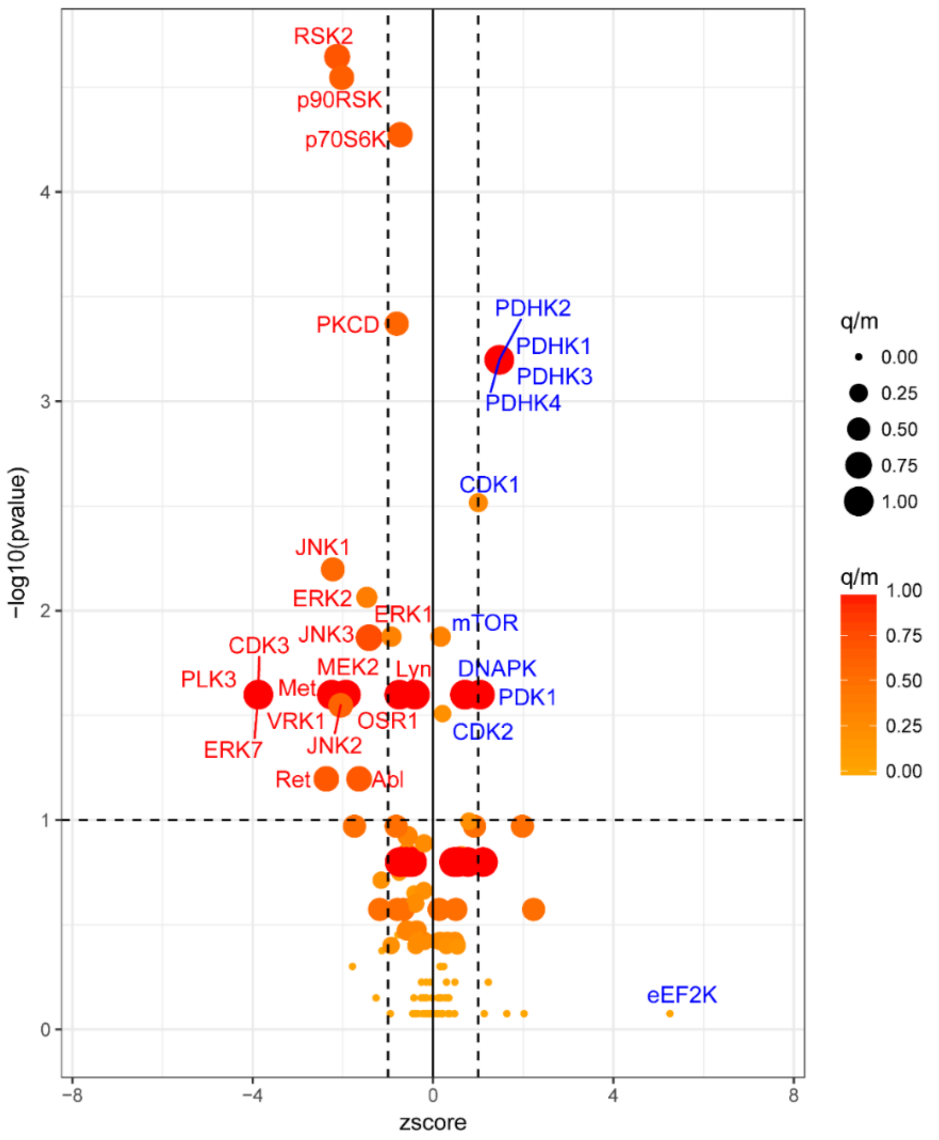

Figure S4. Kinase-Substrate Enrichment Analysis of the phosphoproteomics results. Size and colour intensity of each dot is proportional to the ratio of Ibrutinib-treated samples compared to untreated samples. Kinases that were activated or inhibited in the presence of Ibrutinib are highlighted in blue or red, respectively.

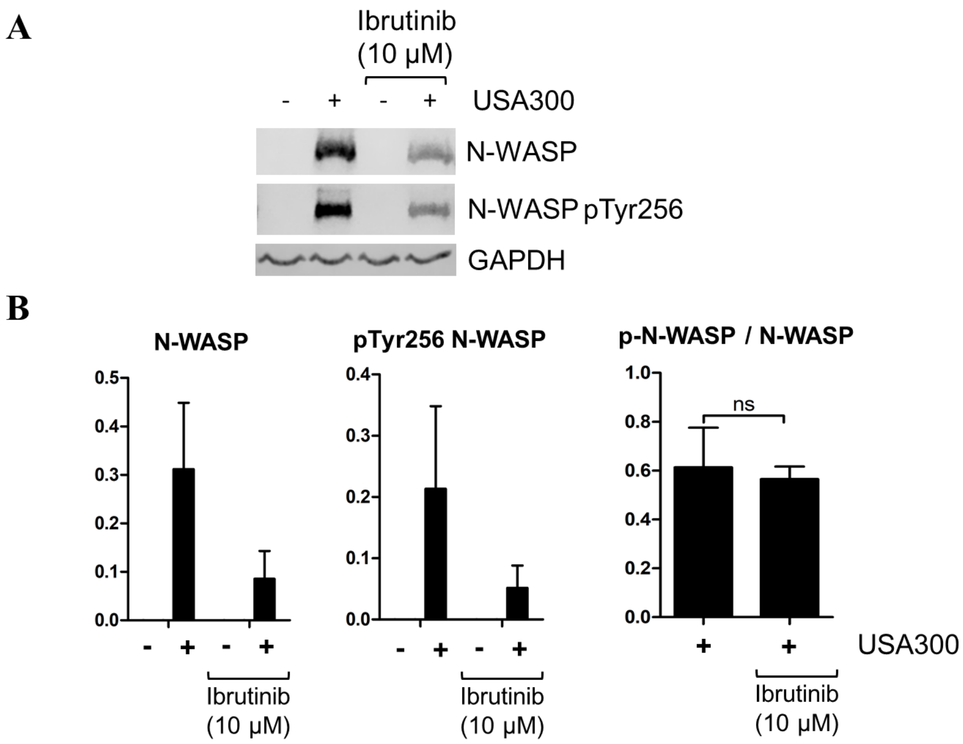

Figure S5. Phosphorylation of N-WASP protein was not significantly affected by Ibrutinib treatment. HeLa cells were infected with *S. aureus* USA300 (MOI 100) for 6 hours in the presence or absence of Ibrutinib. (A) Protein lysates were analysed by Western-blot against N-WASP and phospho-N-WASP. GAPDH antibody was employed as loading control. The cropped blots are used in the figure, and full-length blots are presented in Figure S7. (B) Quantification of N-WASP and phospho-N-WASP levels observed by Western-blot. Data were normalized to GAPDH levels for each condition and are expressed as means ± standard errors (SE) of two independent experiments. Student´s t-tests were performed to validate statistical significance across conditions. (n.s) no statistical significance.

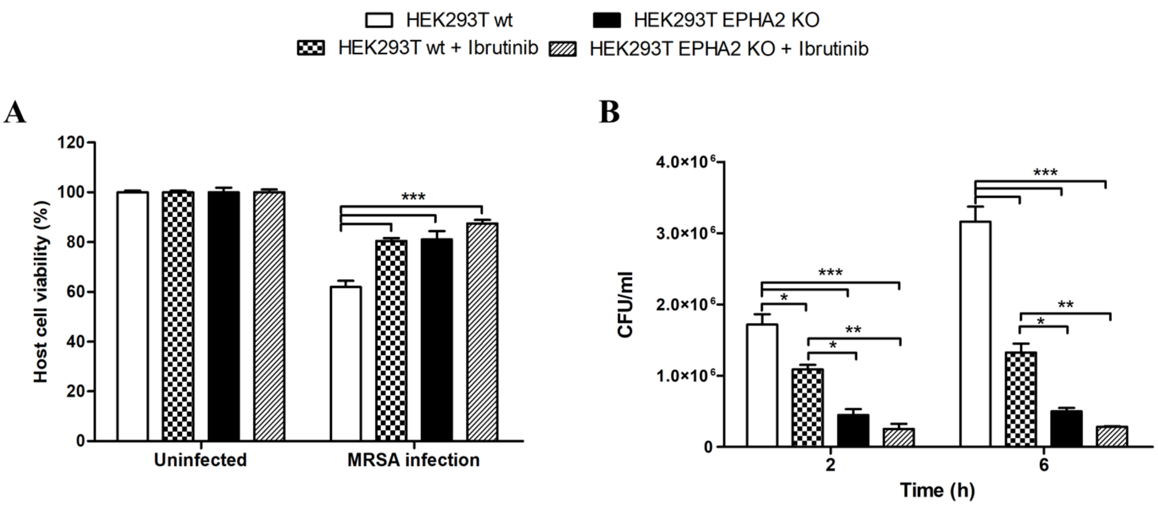

Figure S6. Ibrutinib treatment on HEK293T cells increases cell viability and reduces intracellular *S. aureus* load. HEK293T wt and EPHA2 KO cells were infected with *S. aureus* USA300 (MOI 100; 6 hours) in the presence of DMSO (Mock) or 10 µM of Ibrutinib. (A) Host cell viability was quantified by flow cytometry, using a double annexin V-FITC and PI staining. Cell viability was normalized by the percentage of uninfected and untreated cells. (B) Intracellular MRSA survival was quantified by colony forming unit (CFU) counting after 2 and 6 hours of infection. Data are expressed as means ± standard errors (SE) of three independent experiments performed in duplicates. Two-way ANOVA and Bonferroni *post hoc* tests were performed to validate statistical significance across conditions. p-value ≤0.05 (*); ≤0.01 (**); ≤0.001 (***).

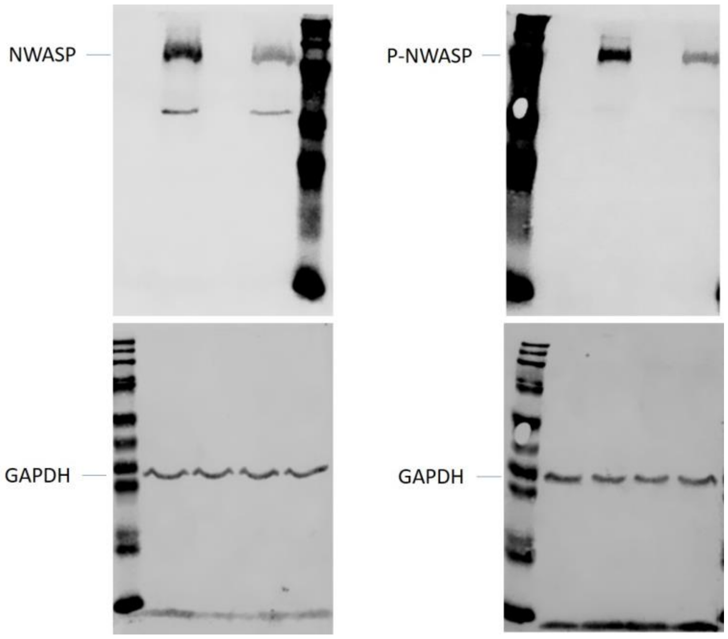

Figure S7. Full length blots of Figure S5A.

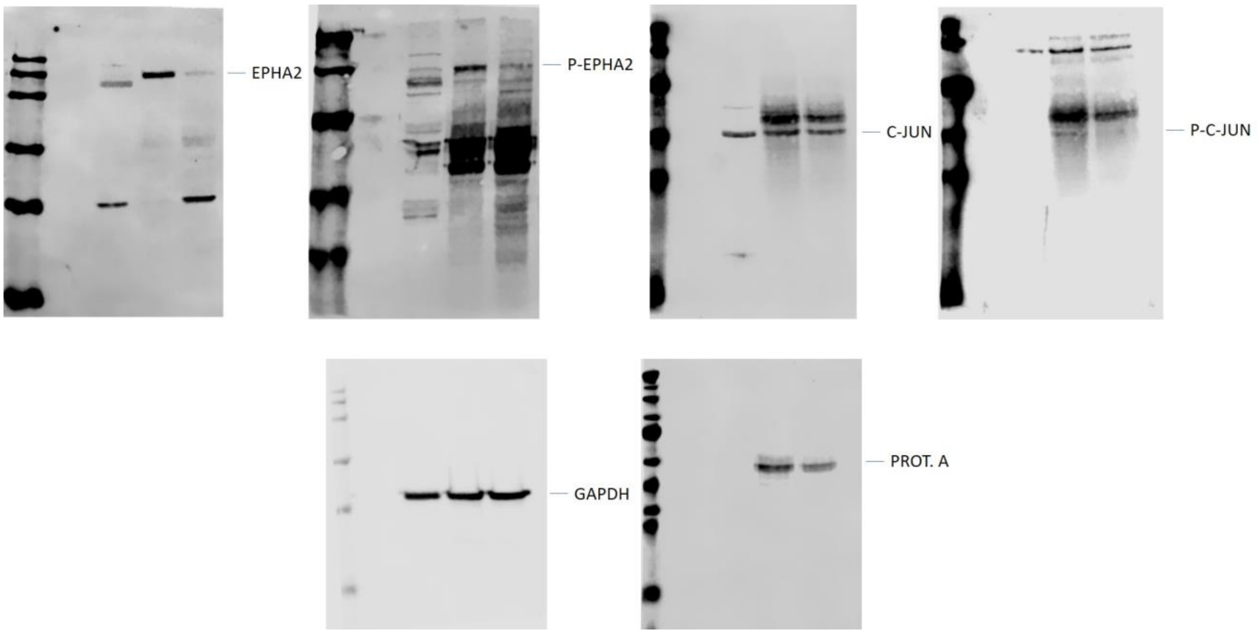

Figure S8. Full length blots of Figure 6A.

59 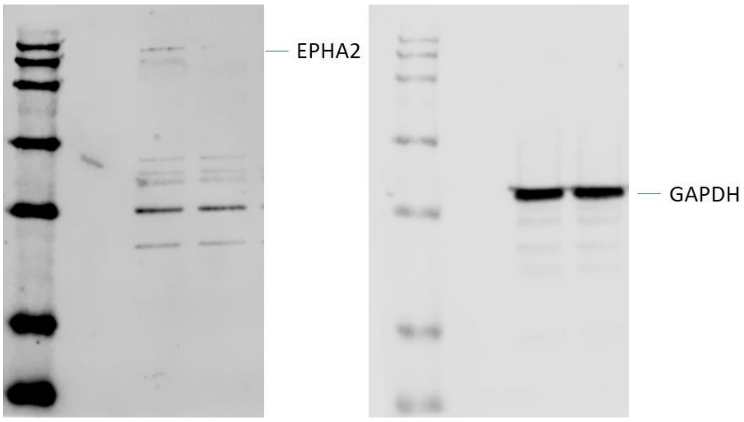

60 **Figure S9. Full length blots of Figure 6B.**

SUPPLEMENTARY TABLES

Drug	Mean	SE	p-value	Mean	SE	p-value
Ibrutinib	93.12	17.17	0.0301	3.58	0.87	0.0073
Dasatinib	99.5	44.5	0.0405	4.84	0.16	0.0078
Crizotinib	98.94	46.62	0.0497	5.91	2.77	0.0083
Mitoxantrone	59.57	25.75	0.6609	8.42	0.72	0.0095
Ceritinib	41.84	3.55	0.2399	10.43	2.51	0.0000
Mitomycin	138.13	7.25	0.0002	10.94	1.34	0.0109
Capecitabine	107.42	29.04	0.0177	10.94	1.34	0.0109
Panobinostat	94.32	37.49	0.0837	12.01	1.59	0.0116
Afatinib	89.41	39.84	0.1310	13.08	4.51	0.0125
Gefitinib	70.54	10.14	0.2308	13.27	0.56	0.0124
Imatinib	98.78	17.61	0.0175	13.46	3.39	0.0127
Clofarabine	81.07	4.90	0.0695	14.52	0.47	0.0133
Mitotane	83.97	7.29	0.0528	15.40	6.41	0.0144
Teniposide	74.61	25.38	0.3498	15.41	6.62	0.0000
Nelarabine	65.57	12.88	0.3077	16.81	7.05	0.0045
Raloxifene	101.86	19.81	0.0150	17.92	4.36	0.0163
Plicamycin	116.02	11.61	0.0023	19.66	5.02	0.0002
Dexrazoxane	60.17	16.65	0.5481	20.20	8.87	0.0060
Belinostat	69.25	30.48	0.3873	20.43	2.30	0.0186
Uracil mustard	60.47	6.94	0.4625	20.83	8.24	0.0062
Vemurafenib	94.41	25.77	0.0648	21.70	8.76	0.0004
Tretinoin	64.65	18.97	0.3964	22.00	12.64	0.0078
Celecoxib	74.46	9.63	0.1548	22.26	10.58	0.0222
Letrozole	74.09	18.63	0.1597	23.05	12.78	0.0084
Idelalisib	107.33	53.60	0.0770	23.33	7.66	0.0228
Sorafenib	109.77	53.11	0.0654	25.27	2.15	0.0245
Trifluridine	64.28	10.40	0.3301	25.49	14.22	0.0106
Olaparib	48.91	7.80	0.8805	26.22	17.61	0.0314
Altretamine	76.06	22.92	0.1644	26.78	14.84	0.0119
Bleomycin sulfate	65.36	17.57	0.5975	27.10	18.76	0.0005
Chlorambucil	97.00	12.98	0.0160	27.79	0.10	0.0282
Fludarabine phosphate	96.06	15.76	0.0206	27.98	3.86	0.0288
Pomalidomide	51.82	6.63	0.9596	29.34	13.03	0.0132
Pentostatin	43.41	12.50	0.5703	30.14	15.30	0.0153
Methotrexate	53.39	12.50	0.8898	30.50	5.91	0.0337
Uridine triacetate	99.62	32.52	0.0426	30.88	13.82	0.0379
Lenalidomide	68.22	20.01	0.2987	31.27	16.56	0.0174
Niraparib hydrochloride	113.25	43.05	0.0302	32.44	3.89	0.0372
Mercaptopurine	73.09	27.40	0.2590	33.15	14.99	0.0186
Thioguanine	97.80	28.93	0.0334	34.67	4.58	0.0156
Temsirolimus	77.37	12.41	0.1698	35.58	10.35	0.0004
Cisplatin	65.38	15.48	0.3377	36.09	16.35	0.0241
Dactinomycin	59.55	7.40	0.8521	37.23	9.33	0.0018
Cytarabine hydrochloride	55.71	16.07	0.7579	37.56	16.97	0.0274
Ribociclib	88.45	28.22	0.0840	39.99	2.27	0.0572

Drug	Mean	SE	p-value	Mean	SE	p-value
Bendamustine	145.72	27.91	0.0008	40.18	6.22	0.0589
Mechlorethamine	62.84	16.37	0.4362	40.32	19.67	0.0368
Carboplatin	109.45	5.44	0.0031	40.56	14.13	0.0655
Axitinib	103.77	38.65	0.0449	41.06	0.66	0.0607
Bortezomib	97.36	24.94	0.0321	42.01	19.11	0.0767
Thiotepa	51.13	18.88	0.9964	43.00	22.76	0.0499
Palbociclib	81.45	10.52	0.0963	44.99	9.60	0.0010
Thalidomide	54.46	10.16	0.8029	45.05	25.84	0.0643
Ponatinib	107.82	10.52	0.0033	45.06	5.14	0.0007
Erlotinib hydrochloride	63.49	18.80	0.4792	45.14	16.31	0.0871
Oxaliplatin	103.68	11.83	0.0075	45.40	14.28	0.0859
Docetaxel	68.48	6.88	0.3887	45.58	3.04	0.0006
Methoxsalen	54.15	12.03	0.8268	46.23	24.06	0.0646
Cobimetinib	55.14	27.27	0.9171	47.23	28.19	0.0120
Aminolevulinic acid	60.95	6.66	0.4387	47.24	21.67	0.0630
Vismodegib	123.78	30.64	0.0054	47.92	16.34	0.1014
Topotecan hydrochloride	127.64	41.48	0.0101	48.60	5.51	0.0949
Pemetrexed, Disodium salt,	69.55	2.43	0.2337	49.17	17.36	0.1101
Pipobroman	55.94	15.97	0.7741	49.67	2.58	0.0998
Cabozantinib	91.36	12.52	0.0242	49.83	11.99	0.0008
Imiquimod	84.91	18.72	0.1029	51.78	14.43	0.0037
Gemcitabine hydrochloride	109.45	23.35	0.0059	51.86	27.93	0.1052
Ifosfamide	66.43	4.50	0.2269	51.99	21.89	0.0864
Irinotecan hydrochloride	83.32	4.05	0.0585	52.01	6.67	0.0017
Rucaparib phosphate	103.26	24.46	0.0182	52.38	8.59	0.1196
Pazopanib hydrochloride	65.83	4.23	0.7443	53.88	8.68	0.0016
Dacarbazine	163.44	23.53	0.0001	54.42	22.83	0.1039
Estramustine phosphate	68.12	11.44	0.4327	54.93	18.37	0.0164
Megestrol acetate	102.06	24.26	0.0201	55.46	22.50	0.1648
Melphalan hydrochloride	115.20	1.93	0.0016	55.52	11.15	0.1451
Tamoxifen citrate	74.55	24.73	0.3449	56.36	29.88	0.0319
Omacetaxine	92.97	21.80	0.0552	56.46	11.47	0.0047
Everolimus	56.16	11.45	0.9448	56.66	6.42	0.0110
Lapatinib	55.84	21.64	0.9396	57.19	15.09	0.0169
Venetoclax	110.18	5.85	0.0017	58.29	16.43	0.0099
Lenvatinib	124.12	2.27	0.0007	58.36	32.46	0.2194
Temozolomide	92.02	9.37	0.0087	60.45	16.46	0.1286
Lomustine	57.68	12.56	0.6359	60.82	29.82	0.1856
Cladribine	97.04	12.58	0.0062	61.21	20.60	0.1493
Decitabine	115.40	21.14	0.0024	61.38	16.22	0.1359
Erismodegib	92.04	40.25	0.0626	62.16	18.05	0.0040
Exemestane	96.46	17.26	0.0114	62.43	20.58	0.1610
Vorinostat	140.89	11.51	0.0000	62.80	24.34	0.1805
Paclitaxel	68.75	7.90	0.3834	62.85	9.20	0.0086
Vinorelbine tartrate	60.78	3.56	0.7706	63.88	3.88	0.0071
Regorafenib	74.30	25.90	0.3632	64.22	12.72	0.0307
Fluorouracil	83.45	37.59	0.1842	64.51	15.92	0.1656

Drug	Mean	SE	p-value	Mean	SE	p-value
Arsenic trioxide	119.92	12.76	0.0004	65.23	26.52	0.2185
Dabrafenib mesylate	114.96	7.27	0.0010	66.14	27.12	0.0610
Ixabepilone	75.36	6.41	0.1764	66.17	32.04	0.0933
Plerixafor	77.44	16.90	0.2028	66.27	25.02	0.0525
Azacitidine	136.35	14.25	0.0001	67.08	19.92	0.2108
Anastrozole	85.42	5.94	0.0172	67.12	23.48	0.2273
Cyclophosphamide	73.29	11.29	0.1195	67.95	17.12	0.2106
Hydroxyurea	90.96	27.83	0.0568	69.12	16.04	0.2222
Floxuridine	62.40	14.30	0.4343	69.52	51.57	0.4179
Trametinib	65.86	21.06	0.6028	69.61	19.37	0.0467
Pralatrexate	72.81	31.93	0.3153	70.98	6.60	0.3172
Osimertinib	78.61	20.66	0.2123	72.02	54.97	0.3076
Amifostine	106.90	22.39	0.0139	72.88	20.04	0.0697
Streptozocin	114.96	26.89	0.0055	74.16	21.16	0.3237
Cabazitaxel	87.69	15.33	0.0605	76.53	12.46	0.0628
Zoledronic acid	51.39	10.20	0.6732	77.25	5.39	0.0479
Procarbazine hydrochloride	115.10	34.60	0.0133	77.43	15.01	0.3587
Triethylenemelamine	54.61	10.97	0.8549	79.17	33.63	0.2470
Carmustine	83.97	16.63	0.0465	80.53	23.99	0.4652
Vincristine sulfate	78.41	2.42	0.1103	80.72	21.01	0.1629
Fulvestrant	80.46	21.57	0.1860	83.22	30.71	0.2963
Allopurinol	108.69	40.74	0.0378	86.60	21.97	0.6044
Bosutinib	95.63	26.56	0.0281	86.88	55.49	0.1253
Etoposide	102.81	8.99	0.0055	87.25	15.77	0.2465
Enzalutamide	73.87	26.47	0.3802	91.54	4.70	0.3893
Carfilzomib	43.78	11.96	0.3491	92.38	32.29	0.5477
Busulfan	53.11	10.92	0.8850	93.11	20.47	0.7855
Alectinib	51.68	8.86	0.2841	93.37	34.10	0.5642
Valrubicin	48.42	6.45	0.5039	94.33	17.34	0.4869
Nilotinib	43.89	4.80	0.7537	95.03	45.99	0.4474
Ixazomib citrate	93.03	14.03	0.0789	97.69	46.66	0.3669
Sirolimus	46.20	15.14	0.4658	101.05	15.19	0.7794
Daunorubicin	70.06	9.43	0.3443	103.79	46.47	0.9578
Vandetanib	96.11	30.67	0.0788	104.60	97.73	0.9911
Vinblastine sulfate	79.02	15.06	0.1586	109.81	16.22	0.7601
Abiraterone	63.92	8.91	0.6105	116.20	23.79	0.5374
Romidepsin	66.63	6.35	0.1377	134.18	16.87	0.7031
Sunitinib	62.87	36.85	0.6078	200.50	25.70	0.0097
Doxorubicin hydrochloride	90.64	6.81	0.0238	222.83	27.38	0.0002
Epirubicin hydrochloride	92.22	8.06	0.0207	405.48	63.93	0.0000
Idarubicin hydrochloride	66.27	23.03	0.6012	5341.22	3598.06	0.0182

Table S1. Results of drug-screening on MRSA infection in HeLa cells. The effect of 133 FDA-approved drugs was tested in HeLa cells after MRSA infection (MOI 100, 6h). The percentage of cell number was normalized to percentage of uninfected cells. The percentage of HeLa cells containing *S.*

aureus USA300-GFP was normalized to the untreated infected cells. Data show mean, standard error and p-value of three biological replicates.

Table S2. Phosphoproteomics results of *S. aureus*-infected HeLa cells under Ibrutinib treatment. The Log2 Fold Change and -Log10 of p-value were calculated for each phosphopeptide identified under Ibrutinib treatment in relation to the untreated control.

Cellular pathway	Number of genes	p-value	Benjamini
Focal adhesion	13	5.10E-08	3.80E-06
ErbB signaling pathway	10	2.10E-08	3.10E-06
Adherens junction	7	1.90E-05	9.30E-04
MAPK signaling pathway	11	2.60E-05	1.00E-03
Regulation of actin cytoeskeleton	10	3.70E-05	1.10E-03

Table S3. Cellular pathways affected by Ibrutinib treatment during MRSA infection. KEGG pathways were analysed employing DAVID Bioinformatics Resources 6.8. Only cellular pathways that were statistically significant after Benjamini correction were included (p-value < 0.01).

Protein	Description	Modification	Log2Fold	p-value
ABL2	Tyrosine-protein kinase ABL2 protein	ABL2(T938)	-1.187	0.0039
ARHGAP35	Rho GTPase-activating protein 35	ARHGAP35 (S1153)	-1.588	1.42E-04
DAXX	Death domain-associated protein 6	DAXX(S495)	-1.304	0.0138
EGFR	Epidermal growth factor receptor protein	EGFR(S695)	-1.422	0.0076
ERBB2	Receptor tyrosine-protein kinase erbB-2 protein	ERBB2(T694)	-1.364	2.27E-04
EPHA2	Ephrin type-A receptor 2 protein	EPHA2(S899); EPHA2(S901)	-2.000	5.57E-04
		EPHA2(S901)	-2.000	5.57E-04
FLNA	Filamin A protein	FLNA(T1739)	-3.122	1.23E-05
		FLNA(S966)	-2.293	1.88E-04
FLNB	Filamin B protein	FLNB(S2487)	-1.499	0.0013
		FLNB(S2113)	-1.168	3.42E-04
JUN	Transcription factor AP-1 protein	JUN(S243)	-3.337	3.83E-05
		JUN(T62)	-3.103	6.84E-08
		JUN(S63)	-2.037	4.56E-05
MAP2K2	Mitogen-activated protein kinase 2	MAP2K2(T230); MAP2K1(T226)	-1.629	0.0092
MAPK3	MAPK3/ERK1 protein	MAPK3(T198); MAPK3(Y204)	-1.390	1.77E-06
		MAPK3(Y204)	-1.390	1.77E-06
NCK1	Cytoplasmic protein NCK1	NCK1(Y105)	-2.159	5.82E-04
PAK2	Serine/Threonine protein kinase PAK 2	PAK2(T21)	-1.378	0.0074
PPP1R12A	Protein phosphatase 1 regulatory subunit 12A	PPP1R12A(T305)	-1.531	1.72E-04
		PPP1R12A(S304)	-1.443	6.26E-05
		PPP1R12A(S299)	-1.294	0.0028
		PPP1R12A(S507)	-2.171	5.94E-04
PTK2	Focal adhesion kinase 1 protein	PTK2(S910)	-1.148	7.21E-04
RPS6KA1	Ribosomal protein S6 kinase alpha-1	RPS6KA1(S732)	-1.189	1.02E-06
SHC1	SHC-transforming protein 1	SHC1(S426)	-2.853	9.71E-06
		SHC1(Y427)	-2.853	9.71E-06
TGFBR2	TGF-beta receptor type-2 protein	TGFBR2(S352)	-1.453	2.48E-04
TLN1	Talin 1 protein	TLN1(S1225)	-1.595	4.09E-05
		TLN1(S1227)	-3.955	0.0439
WASL	Neural Wiskott-Aldrich syndrome protein	WASL(Y256)	-1.112	3.16E-05

Table S4. List of proteins involved in KEGG pathways affected by Ibrutinib treatment during *S. aureus* infection.
